# Computational Methods to Predict Conformational B-Cell Epitopes

**DOI:** 10.3390/biom14080983

**Published:** 2024-08-10

**Authors:** M. Carroll, E. Rosenbaum, R. Viswanathan

**Affiliations:** Department of Chemistry and Biochemistry, Yeshiva College, Yeshiva University, New York, NY 10033, USA; mcarrol2@mail.yu.edu (M.C.); esrosen2@mail.yu.edu (E.R.)

**Keywords:** B-cell epitopes, conformational epitopes, computational identification of epitopes

## Abstract

Accurate computational prediction of B-cell epitopes can greatly enhance biomedical research and rapidly advance efforts to develop therapeutics, monoclonal antibodies, vaccines, and immunodiagnostic reagents. Previous research efforts have primarily focused on the development of computational methods to predict linear epitopes rather than conformational epitopes; however, the latter is much more biologically predominant. Several conformational B-cell epitope prediction methods have recently been published, but their predictive performances are weak. Here, we present a review of the latest computational methods and assess their performances on a diverse test set of 29 non-redundant unbound antigen structures. Our results demonstrate that ISPIPab performs better than most methods and compares favorably with other recent antigen-specific methods. Finally, we suggest new strategies and opportunities to improve computational predictions of conformational B-cell epitopes.

## 1. Introduction

**Figure 1:**
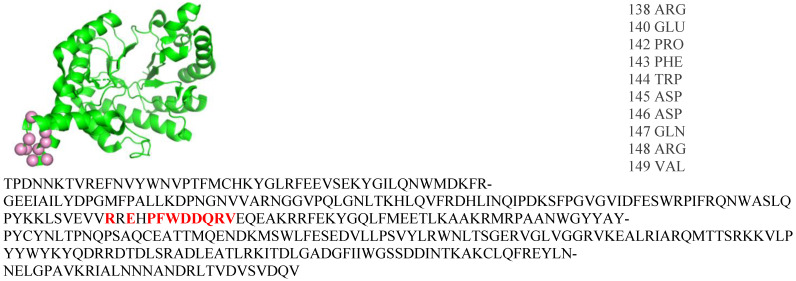
Shown at the top left is the unbound structure of hyaluron-glucosaminidase (PDB ID: 1FCQ) with the epitope residues shown as pink spheres. The epitope residue names and numbers are listed on the top right and shown bolded in red within the FASTA sequence. Based on the sequential proximity, the epitope is linear.

**Figure 2:**
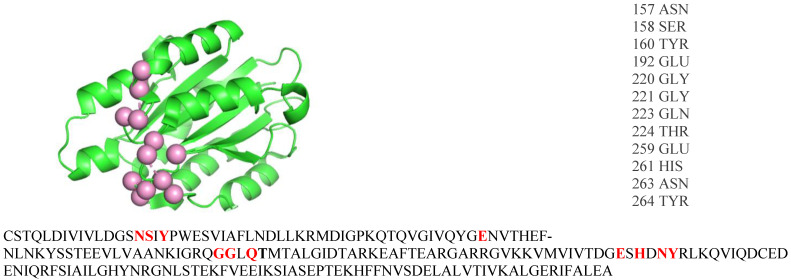
Shown at the top left is the unbound structure of integrin alpha-1 (PDB ID: 1CK4) with the epitope residues shown as pink spheres. The epitope residue names and numbers are listed on the top right and shown bolded in red within the FASTA sequence. Since the residues are only spatially proximal, the epitope is conformational.

B-cell epitopes (BCE) are specific regions on the surfaces of protein antigens that are recognized by antibodies. BCE are generally classified as either linear or conformational. Linear BCE are sequentially consistent with the primary structure of the antigen (Figure 1), while conformational or discontinuous BCE are spatially proximal within the three-dimensional structure of the antigen (Figure 2). However, greater than 90% of BCE are presumed to be discontinuous in nature [1], and a recent analysis found that only 4% are truly linear [2].

Accurate mapping of BCE is critical in biomedical research to develop therapeutics, antibodies, peptide-based vaccines, and immunodiagnostic reagents [3,4,5]. Experimental methods used to map epitopes can be grouped into either structural or functional studies [6]. X-ray crystallography and NMR are the two most reliable structural methods for BCE mapping [7,8]. However, these methods are expensive, time-consuming, and inefficient [9]. Functional studies have proven to be relatively more cost-effective and efficient and include new techniques such as mimotope analysis and display technologies [10,11]. Other functional mapping studies include peptide screenings for antibody binding as well as antibody–antigen mutant reactivity testing [12].

Computational approaches present complementary techniques to experimental methods for identifying BCE, and their complexities and accuracy have gradually improved in recent decades [9]. Two distinct classes of BCE prediction methods have been developed, and both use a variety of machine learning approaches to integrate several input features [13]. First, linear BCE prediction methods are based on amino acid properties, including antigenicity, flexibility, hydrophilicity, solvent accessibility, and secondary structure [14,15,16,17,18,19]. Second, discontinuous BCE prediction methods integrate structural and surface patch features, including hydrophilicity scale, epitopic residue propensity score, residue protrusion index, and residue compactness, among several other input vectors [1,20,21,22]. However, most available BCE prediction methods are designed for linear epitope prediction due to their simpler computational development and sole reliance on primary structural data, which have greater availability [13]. Despite the much greater biological prevalence of discontinuous as opposed to linear BCE, the development of prediction methods for the former have received significantly less attention [6]. Previous work has found that structure-based methods are superior to sequence-based methods [23]. However, currently available computational BCE prediction methods continue to lag, as they are largely inaccurate and their development is limited by the availability of resolved antigen–antibody complex structures as well as the ongoing challenges in extracting discontinuous epitopes for antibody development [24,25,26].

Further distinctions among linear and discontinuous BCE prediction method classes exist when considering the requirements for antibody-specific or antibody-independent predictions. The development of antibody-independent BCE predictors has received greater attention as compared to antibody-specific BCE predictors, and a recent comparison asserts the former class boasts greater overall performance, according to several statistical measures, but lower precision [27,28]. While antibody-specific approaches are more targeted and controlled, they require that the paired antibody be successfully screened and identified. However, prior knowledge of the cognate antibody is not always known and thus requires significant screening. Current antibody-specific methods cannot efficiently screen the approximately 10^16^–10^18^ possible antibodies, rendering this method inapplicable in many cases which have limited background biological information [28,29]. Furthermore, conformational changes are expected to different degrees during antigen–antibody binding, and an understanding of these changes is only available with the resolved bound antigen–antibody complex, which is also not commonly available [30]. Overall, this underscores the need for the development of generic methods that can accurately predict BCE, especially those solely using the unbound antigen structure.

**Figure 3:**
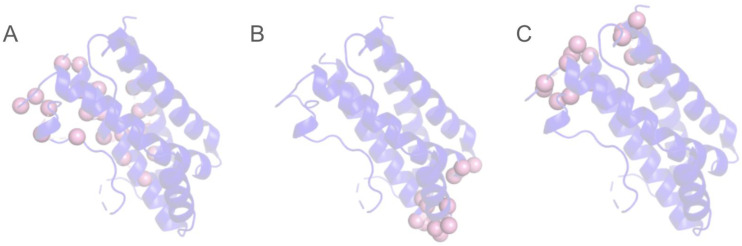
Three distinct experimentally determined epitopes for human interleukin 6 (PDB ID: 1ALU) when complexed with (**A**) olokizumab, (**B**) camelid, and (**C**) Llama Fab fragment 68F2 antibodies with the epitope residues shown as pink spheres.

BCE prediction faces an additional challenge, as antibody–antigen interactions can occur across multiple regions on the surface of an antigen of interest (Figure 3). Currently, it remains unknown whether any region of an antigen’s surface can theoretically be targeted by antibodies [28]. However, it is certain that specific regions are targeted much more favorably during immune responses, an immunological phenomenon known as epitope immunodominance [31,32]. The ability to accurately predict immunodominant and subdominant BCE has significant applicability in biomedical research to develop antibodies or vaccines that target these sites. Therefore, the consideration of multiple epitopes should be a critical factor in the development of computational BCE prediction methods. Zhang et al. developed CBEP, which uses primary sequence features as well as a K-means clustering algorithm to identify multiple epitopes using residue spatial locations and a threshold parameter [33]. Additionally, the method developed by Ren et al. uses sequential information and various patterns of propensities for BCE prediction alongside a clustering method with a pre-defined optimal cutoff distance of 6 Å between any pair of residues belonging to the same cluster to identify multiple epitopes [34]. Our recent work combines our computational method, ISPIPab, with hierarchical clustering to predict multiple epitopes. After calculating the pairwise distances between the geometric centers of predicted epitopal residues, a dendrogram is constructed. The optimal number of clusters for each antigen corresponds to the number of vertical lines in the dendrogram cut by a horizontal line that can traverse the maximum distance vertically without intersecting a cluster. Thus, the number of distinct predicted epitopes is determined dynamically for each antigen [35].

In recent years, several discontinuous BCE prediction methods have been published. Consequently, there are calls for independent evaluations of the available methods with an emphasis on including confusion matrix-based performance metrics, such as F1-score and MCC, to better assess predictive performance [24,28,36,37,38,39]. This review compares the performance of the latest computational methods for the prediction of conformational BCE epitopes on a set of antigens selected with ≤30% sequence identity and to span a range of protein lengths and CATH protein family classifications.

## 2. Materials and Methods

### 2.1. Databases for BCE Epitopes

Experimental protein data have grown tremendously in recent decades, including B-cell epitope data, which are critical for the development of well-trained and accurate BCE prediction methods [40]. Several web-based databases host relevant BCE data, which are both automatically and manually curated. The Protein Data Bank (PDB), maintained by the RCSB, stores three-dimensional structures of proteins and complexes derived from X-ray crystallography, NMR, and cryo-EM studies with over 200,000 entries [41]. The automated web-based summary, SACS, catalogs antigen–antibody complexes and information extracted from the PDB [42]. The Immune Epitope Database (IEDB), managed by the La Jolla Institute for Allergy and Immunology, is the most popular and used BCE database. The IEDB catalogs experimentally determined linear and discontinuous epitopes as well as T-cell epitopes studies and MHC assays from published literature. The IEDB also hosts several epitope prediction methods and analysis tools. At this moment, the IEDB hosts data from over 1.4 million B-cell assays and 1.6 million peptidic epitopes [43,44]. BciPep, established by the Institute of Microbial Technology, catalogs experimentally determined linear BCE data. BciPep currently lists thousands of epitopes with a focus on those from various pathogenic organisms, including viruses, fungi, bacteria, and protozoa. The epitopes are of varying immunogenicity and compiled from the literature as well as other public databases [45]. The Conformational Epitope Database (CED), established by the Institute for Chemical Research, compiles data of discontinuous epitopes. The CED is manually curated with limited entries and features only high-quality conformational epitopes described in the literature. Entries include source antigens, cognate antibodies, and relevant immunological properties, and epitope locations which can be visualized within the interface [46]. Additionally, Epitome extracts epitopes from all known resolved antigen–antibody complexes. The database includes descriptions of the epitopal residues as well as sequential and structural environments, and can be viewed through a Jmol interface [47]. AntiJen, established by the Edward Jenner Institute for Vaccine Research, is another useful database that integrates kinetic, thermodynamic, functional, and cellular data within the context of immunology and vaccinology. AntiJen includes more than 31,000 comprehensive entries from experimental studies and includes data of peptide binding to MHC ligands, TCR–MHC complexes, T-cell epitopes, TAP, B-cell epitope molecules, and immunological protein–protein interactions [48]. Finally, the HIV Molecular Immunology Database, established by the Los Alamos National Laboratory, catalogs and annotates HIV epitopes compiled from the literature with over 10,000 HIV-specific B-cell and T-cell assays. The entries include descriptions of escape mutations, antibody sequences, TCR usage, cross-reactivity, and associations between immune responses and rates of therapy and progression, among other information [49].

### 2.2. Dataset Used to Compare Performance

Jespersen et al. curated a dataset of 335 antibody–antigen complexes with experimentally identified epitope residues, which includes entries from the IEDB and PDB. The complexes include structures with <3 Å resolution, antigens with greater than 60 residues, and B-cell heavy-light chain receptors. The authors removed redundancies by clustering the antibodies and antigens at 90% and 70% sequence identity thresholds, respectively, which returned 202 antibody–antigen clusters [50].

We expanded our dataset by searching for antigen–antibody complexes in the SACS database that included antigens between 100 and 450 residues, resolutions of <3 Å, and entries published after 2005. This returned 2196 complexes, and the BCE were determined using the CSU program, which employed a cutoff value of 4.0 Å between any heavy atom in a residue in the antigen and a heavy atom in the antibody of the complex, and established a legitimate contact type according to CSU [51].

**Figure 4:**
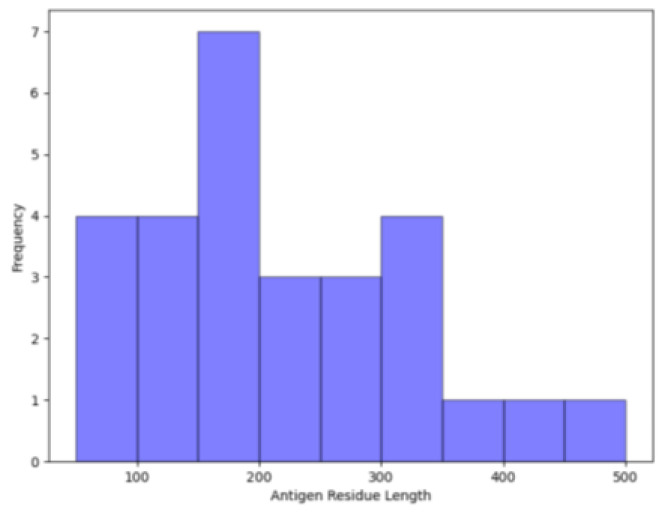
Distribution of the number of residues among the 29 test set antigens. One antigen with 723 residues is not included in the figure.

**Figure 5:**
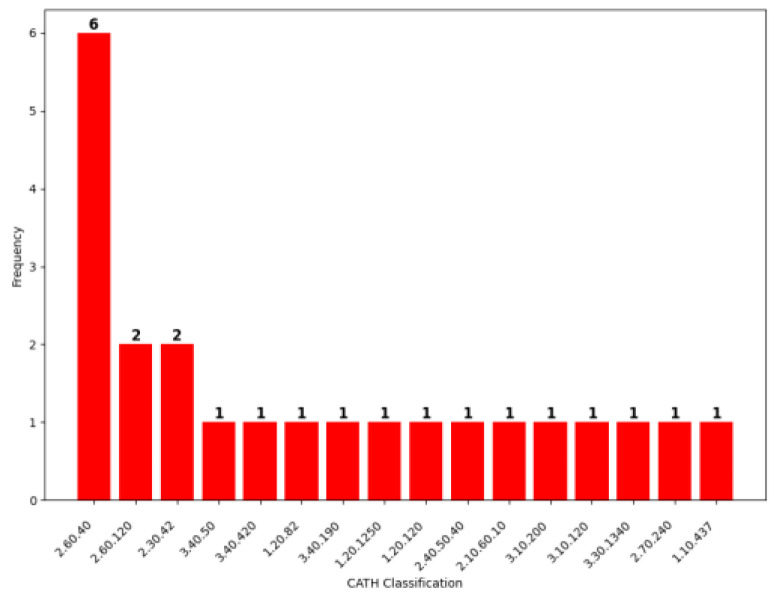
CATH family classifications of test set antigens. CATH classifications were not available for all 29 antigens.

Since we sought to assess predictive performance on unbound antigen structures, we searched for analogous unbound antigens in the PDB that shared >95% sequence identity to the bound complexed antigens. We further refined the antigen datasets by removing redundancy at a sequence identity threshold of ≤30%. Collectively, this returned 76 and 35 uncomplexed monomer antigens from the Jespersen et al. and SACS datasets, respectively, for a total of 111 unbound antigens. The epitope residues on the unbound antigen structures were identified by sequentially aligning them to those determined in their analogous bound antigen structures. A total of 82 of these antigens were used to train our ISPIPab method, and the remaining 29 antigens were used for testing. These 29 non-redundant antigens are of varying lengths (Figure 4) and represent a variety of CATH classifications (Figure 5) [52]. The PDB IDs of these test set antigens is included in Table S1.

### 2.3. Computational Methods Evaluated for Epitope Prediction

As described above, our evaluation tested the predictive performances of recently developed computational methods, including those developed specifically for discontinuous BCE prediction, and all methods were antibody or partner independent. The default parameter settings for these methods, as set in their respective web servers, were used to perform the computational epitope predictions.

#### 2.3.1. ISPIPab

ISPIPab [35] is a recently developed meta-method that integrates the predictions of SPPIDER [53], ISPRED4 [54], and DockPred [55] using XGBoost to predict B-cell epitopes. ISPIPab is based on the hypothesis that computational BCE prediction can be improved through integrating independent classifiers that leverage structure-based, template-free, and docking-based approaches [56]. As this method is implemented using XGBoost, the parameters that characterize the collection of trees within the forest, including the maximum depth allowed for each tree and a tree pruning parameter denoted as α—responsible for selecting the subtree that minimizes the cost complexity measure—were fine-tuned to identify the optimal model fit. To maximize the model’s performance, 100 trees, 10 levels, and a zero pruning parameter were used. In XGBoost, the successive trees “learn” from previous trees through the use of a loss function and regularization parameter. After the trees undergo training with the training set antigens, the model categorizes the residues in the test set antigens by assigning them to specific terminal nodes (leaves) within each tree of the forest. Utilizing information obtained during the training phase, the model computes the probability of a residue being epitopal for each terminal node in every tree. This process yields a probability value for each terminal node in each tree of the random forest. Ultimately, the overall probability of a residue being epitopal is determined by averaging the probabilities associated with all terminal nodes across the entire forest to which the test antigen residue has been classified. We demonstrated that ISPIPab’s BCE predictive performance outperformed that of its individual classifiers, comparable meta-methods, and BCE-specific prediction methods. Finally, ISPIPab includes a hierarchical clustering methodology that clusters predicted epitope residues into potential epitope regions, thus accounting for the presence of multiple epitopes. This feature first selects the appropriate number of top-performing residues according to their epitopal probability scores determined by ISPIPab and the dynamic cutoff. Next, hierarchical clustering is performed by determining the Euclidean distance between these selected residues’ geometric centers, thus assigning these residues to distinct predicted epitope regions based on an optimal cluster size. We demonstrated that ISPIPab accurately predicts experimentally known epitopes as well as putative epitopes that may not yet be experimentally determined.

#### 2.3.2. EPSVR

Liang et al. describes the development of the discontinuous BCE prediction method EPSVR. EPSVR uses the three-dimensional antigen structure as its input and integrates six scoring terms—including residue epitope propensity, conservation score, side chain energy score, contact number, surface planarity score, and secondary structure composition—through support vector regression implemented using the LIBSVM package [57]. An independent evaluation using a test set of 19 unbound antigen structures from CED with a sequence identity threshold of <35% found that EPSVR had greater percent accuracy (24.7%) than PEPITO (17.0%) [22], SEPPA (17.2%) [20], EPCES (17.8%) [38], EPITOPIA (18.8%) [58], ElliPro (14.3%) [21], and DiscoTope 1.2 (15.5%) [1]. Another review found that EPSVR had a greater AUC value (0.606) compared to SEPPA (0.589), DiscoTope 1.2 (0.579), EPITOPIA (0.572), and EPCES (0.569) [59].

#### 2.3.3. Epitope3D

Silva et al. describes the development of epitope3D, a discontinuous BCE prediction method. The authors curated a dataset of antibody–antigen complexes using the ANARCI tool [60] with antigens of at least 25 residues and compiled 1351 complexes. Unbound antigens were identified through >70% sequence similarity to the bound antigen, and epitopal residues were aligned through several steps. The antigens were clustered using CD-HIT [61] with a 70% similarity cutoff, which returned 245 unbound antigen structures, 180 of which were used for training. Epitope3D leverages the concept of graph-based signatures that model epitope and non-epitope regions as graphs, thereby extracting distance patterns to develop the method. The authors also presented an independent test set of 45 unbound antigens and concluded that epitope3D significantly outperformed SEPPA 3.0 [62], BepiPred 2.0 [63], DiscoTope 2.0 [23], and ElliPro according to the F1-score, MCC, and BACC metrics [37].

#### 2.3.4. BepiPred

Clifford et al. describes the development of BepiPred 3.0, a sequence-based BCE prediction method for both linear and discontinuous epitopes. The authors curated their BP3 dataset that includes 1466 antigen–antibody complexes from the PDB with crystal structures of resolutions < 3 Å, R-factor < 0.3, and antigens of more than 38 residues. The dataset was further refined using an epitope collapse strategy and clustering at 70% sequence identity using MMseqs2 [64]. BepiPred 3.0 exploits protein language model embeddings to accurately predict local and global protein structural features using only amino acid sequences. For comparative evaluation, BepiPred 3.0 was retrained using a five-fold cross-validation setup on 200 antigens and tested on the same 45-antigen test set curated by Silva et al. BepiPred 3.0 had the best ROC-AUC performance (0.71) as compared to epitope3D (0.59), BepiPred 2.0 (0.58), SEPPA 3.0 (0.55), DiscoTope 2.0 (0.51), and ElliPro (0.49). The authors concluded that protein language models can improve BCE prediction, and their sequence-based method outperforms structure-based methods, even in cases of discontinuous epitopes [65].

#### 2.3.5. SEPPA

Zhou et al. describes the development of SEPPA 3.0, which uses three-dimensional antigen structures to perform BCE predictions. SEPPA 2.0 introduced relative ASA preference of unit patch and consolidated amino acid index features to develop a logistic regression model alongside the unit-triangle propensity and clustering coefficient classification parameters introduced in the original model [66]. SEPPA 3.0 improves its predictive performance by updating its training dataset with 767 bound antigen structures and incorporating additional features to enhance the prediction of N-linked glycoprotein antigens. According to the authors’ evaluation, SEPPA 3.0 had greater ROC-AUC values (0.749) than EPITOPIA (0.664), DiscoTope 2.0 (0.660), PEPITO (0.676), CBTOPE (0.541) [67], SEPPA 2.0 (0.651), and BepiPred 2.0 (0.591) as well as balanced accuracy for BCE predictions on general and N-linked glycoprotein antigens.

#### 2.3.6. DiscoTope

DiscoTope, which was first released in 2006, predicts BCE using three-dimensional antigen structures. DiscoTope 2.0 integrates half-sphere exposure surface measure, spatial neighborhood, and amino acid composition features and was found to be a top-performing method in a recent review [28]. DiscoTope 3.0, which was recently released, features inverse folding structure representations and a positive unlabeled learning strategy that are adapted for BCE prediction on both solved and predicted structures [68]. DiscoTope shares a similar training set to that of BepiPred 3.0, using 582 antibody–antigen complexes with <3 Å resolution and an R-factor < 0.3. Using MMseqs2, redundancies to the BepiPred 3.0 test set were removed at a 20% sequence identity threshold, and antigen sequences were clustered at a 50% sequence identity threshold. This resulted in 1125 and 281 antigen clusters that were selected for training and validation, respectively. On an independent test set of 24 non-redundant antigens, DiscoTope 3.0 was shown to outperform BepiPred 3.0, ScanNet [69], and SEMA [70].

We also tested the application of generic protein interface prediction methods to BCE prediction, as our previous work demonstrated that they could outperform BCE-specific prediction methods.

#### 2.3.7. VORFFIP

VORFFIP is a strong-performing complex structure-based protein interface prediction method that integrates heterogeneous data—including various residue level structural and energetic features, evolutionary sequence conservation, and crystallographic B-factors—using a two-step random forest classifier to assign residues with interfacial probability scores. Trained and validated on unbound proteins from the Benchmark 3.0 dataset, VORFFIP was found to compare favorably against other reported protein interface prediction methods [71].

#### 2.3.8. Spatom

Spatom, recently developed by Wu et al., is a structure-based protein interface prediction method. Spatom first defines a weighted digraph for a protein structure to characterize the spatial contacts of residues, followed by the development of a weighted digraph convolution to aggregate both spatial local and global information. Finally, the method adds an improved graph attention layer to drive the predicted sites to form more continuous interfaces [72]. Spatom was trained and tested using the Protein–Protein Docking Benchmark 5.5, which provides 542 unbound structures from 271 diverse protein–protein complexes, including antibody–antigen complexes [73]. On a test set of 80 proteins, Spatom was shown to outperform DeepPPISP [74], SPPIDER, MaSIF-site [75], GraphPPIS [76], and ScanNet. Notably, the authors presented several examples of accurate BCE predictions on antigen structures to demonstrate the versatility of the method.

#### 2.3.9. Performance Assessment

The aforementioned computational methods return interface or epitope probability scores (*p*) that range between 0 and 1 for each residue in each antigen. Since we do not have prior knowledge of the number of epitopal residues located on any given antigen, a dynamic threshold was implemented to determine the threshold for the number of top-scoring residues considered as epitopal in each antigen. The dynamic threshold, N, was calculated using the following equation proposed by Zhang et al.: *N* = 6.1 *R* ^0.3^, where R is the number of surface-exposed residues on the antigen [77]. Through the dynamic threshold, we returned a set of predicted epitope residues per antigen for each computational method that was tested.

Using the dynamic threshold to determine the predicted epitopal residues and experimental data or CSU, which uses the experimental structures of an antigen–antibody complex, to determine the true epitopal residues (annotated residues), the elements of the confusion matrix—true positive, true negative, false positive, and false negative—were calculated. The predictive performance of each method was assessed using the F1-score, Matthew’s correlation coefficient (MCC), and the areas under the receiver operating characteristic (ROC-AUC) and precision-recall (PR-AUC) curves. These methods of statistical analysis best capture the overall computational performance of the tested methods, as both threshold-dependent (F1-score and MCC) as well as threshold-independent (ROC-AUC and PR-AUC) metrics are scored and are the standard metrics to assess the performance of classification models. The F1-score ranges from 0 to 1 and balances precision and recall evenly. The MCC ranges from −1 to 1, accounts for all four elements of the confusion matrix, and provides a more balanced view of performance, especially in imbalanced datasets, which is the case here, as only a small percentage of a given antigen’s residues comprise the epitope. ROC-AUC evaluates performance across all possible thresholds, and PR-AUC focuses on performance in the positive class. The ROC curves were generated by plotting the true positive rate (TPR) against the false positive rate (FPR) for different threshold values of *p*, ranging from 0 to 1. The PR curves were generated by plotting the precision against recall for different threshold values of *p*, ranging from 0 to 1. The PR-AUC metric is further helpful, as it is more sensitive to class imbalances than the ROC-AUC metric. The ROC and PR AUCs were calculated in Python scikit using the trapezoidal method. Table 1 provides the formulas for the calculation of these metrics.

Finally, to determine if the differences in these metrics between various methods were statistically significant, we could not simply use the standard deviation, as the F1-scores and MCC were not normally distributed according to the nonparametric Kolmogorov–Smirnov (KS) single-sample test. Using the KS two-sample test, the F1-scores and MCC were considered to be statistically significant if the *p*-value of the test was <0.05.

## 3. Results

Our test set of 29 unbound antigens only had one experimentally identified epitope, and the antigens were diverse, belonging to several different protein folds, were of varying lengths, and the overall set had a sequence identity threshold of ≤30%. We predicted the BCE on these antigens using ISPIPab as well as DiscoTope 3.0, DiscoTope 2.0, EPSVR, SEPPA 3.0, BepiPred 3.0, epitope3D, VORFFIP, and Spatom. Each of these methods were accessed via their respective web servers. A dynamic threshold was implemented for each antigen to determine the number of residues considered as epitopal for each method, and performance was evaluated using F1-score, MCC, ROC-AUC, and PR-AUC (Table 2).

The F1-scores and MCC do not follow a normal distribution as tested by a single-sample KS test. ISPIPab has the greatest average F1-score (0.312) and MCC (0.230) compared to all other methods that were tested. ISPIPab’s F1-score and MCC performance were greater than those of SEPPA, VORFFIP, DiscoTope 2.0, EPSVR, Spatom, and epitope3D and was shown to be statistically significant, with *p*-values < 0.05, using the two-sample KS test. However, according to the F1-score metric, ISPIPab’s performance as compared to BepiPred 3.0 and DiscoTope 3.0 was not statistically significant, both with *p*-values of 0.29. Similarly, ISPIPab’s MCC performance was not statistically significant as compared to BepiPred 3.0 and DiscoTope 3.0, with *p*-values of 0.19 and 0.11, respectively.

**Figure 6:**
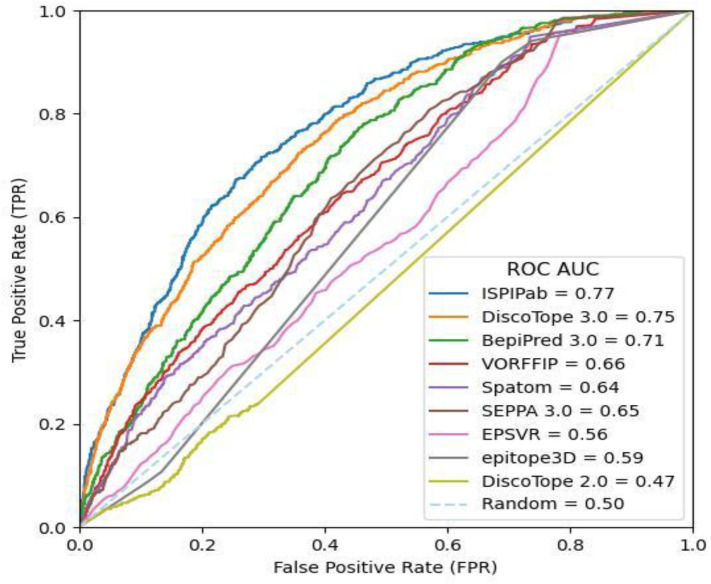
Comparison of ROC curves for the different prediction algorithms.

**Figure 7:**
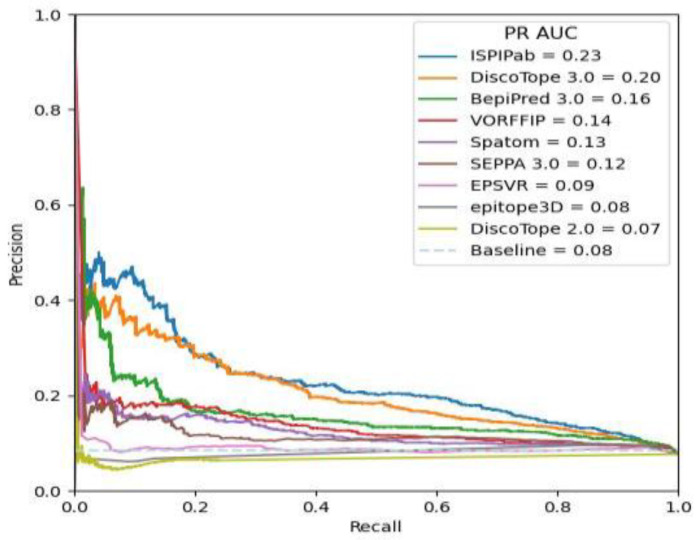
Comparison of PR curves for the different prediction algorithms.

ISPIPab also outperformed the tested methods according to the ROC-AUC and PR-AUC metrics. The ROC and PR curves obtained using the different methods are compared with the results from ISPIPab in Figure 6 and Figure 7, respectively. ISPIPab’s ROC-AUC was 0.77, which is slightly greater than that of DiscoTope 3.0, which had an ROC-AUC of 0.75. The only method to perform worse than random was DiscoTope 2.0, which had an ROC-AUC value of 0.47. These trends were similarly evident according to the PR-AUC metric. ISPIPab had the greater AUC (0.23), followed by DiscoTope 3.0 (0.20) and BepiPred 3.0 (0.16), with only DiscoTope 2.0 performing worse than random.

Notably, VORFFIP, a generic protein interface prediction method, had greater predictive performance than SEPPA 3.0, epitope3D, DiscoTope 2.0, and EPSVR according to all four metrics. Spatom, another generic method, had greater performance than epitope3D and DiscoTope 2.0 according to all four metrics. Spatom also had greater ROC-AUC and PR-AUC values than SEPPA 3.0 but a lower average F1-score and MCC. Collectively, these results suggest that generic protein interface methods can be applied to BCE prediction and more reliably predict BCE than several BCE-specific prediction methods. Thus, future evaluations of computational BCE prediction should include the latest generations of generic protein interface prediction methods to better survey the progress and state of computational BCE prediction.

Moreover, ISPIPab, which integrates the generic methods ISPRED4, DockPred, and SPPIDER, also demonstrates superior performance among the tested methods. This strengthens our previous hypothesis that a meta-method based upon orthogonal methods that each depend on different structural properties of proteins improves interface predictive performance as compared to any individual classifier alone across a diverse class of proteins. Meta-learning approaches have previously been applied successfully in the area of BCE prediction [57,78]. Thus, considering the publication of several BCE prediction methods in recent years, efforts to develop meta-methods based upon BCE prediction tools should be renewed and compared to generic meta-methods in an effort to improve BCE predictive performance.

## 4. Conclusions

This review included several differences from other reviews previously published and builds upon their progress. First, our review focuses on discontinuous rather than linear BCE prediction methods. Second, other reviews test predictions on the bound antigen structure, while we test predictions on the unbound structures, which better assess the genericity and applicability of the methods. Third, our test dataset features a lower sequence identity threshold of ≤30% with antigens of varying lengths and different CATH classifications, thus better assessing the methods’ predictive performances on a diverse set of antigens with reduced biases. And fourth, our evaluation employs a dynamic threshold, which determines the number of predicted epitope residues on the basis of the number of surface-exposed residues for each antigen. Therefore, the number of predicted epitopal residues is independent of the tested method and is determined dynamically based on the structure of the antigen of interest. The results demonstrate that ISPIPab performs better than most methods and compares favorably with recent antigen-specific methods we surveyed according to F1-score, MCC, ROC-AUC, and PR-AUC. Furthermore, we demonstrated that other generic protein interface prediction methods also outperformed some BCE-specific methods.

### 4.1. Inaccessible Web Servers

While this review evaluated a variety of recently published computational BCE prediction methods, there are several other reported methods that have previously been shown to be robust but whose web servers are currently unavailable and thus could not be evaluated. These methods include CEP [79], PEASE [80], EpiPred [81], EPITOPIA, PEPOP [82], CBEP, EPMeta, and BEpro/PEPITO, and the last three methods are particularly notable. A recent review found BEpro/PEPITO was a top-performing method, comparable to DiscoTope 2.0, yet it remains inaccessible [28]. Additionally, EPMeta and CBEP are among the few BCE meta-learning and clustering methods, respectively, published in recent years. As predictive performance of conformational BCE is notoriously weak, new strategies are especially needed [83]. The inaccessibility of all these methods, therefore, presents setbacks to promising areas, such as meta-learning and clustering, that can advance the state of computational BCE prediction.

### 4.2. Limited Availability of Complexed Antibody–Antigen Structures

While we sought to compare the predictive performance of various computational approaches, our conclusions are limited by the size of our test set, which comprises 29 non-redundant unbound antigens. However, our test set antigens spans a range of CATH classifications and lengths, and the test set size is comparable to those of other antigen-specific methods, such as EPSVR [57], epitope3D [37], and DiscoTope 3.0 [68]. As discussed above, the crystallization of antibody–antigen complexes and the mapping of BCE is a costly and time-consuming process. Currently available complexes present just a fraction of all possible antigens and their cognate antibodies [65]. The limited availability of experimentally solved structures presents challenges in improving BCE predictive performance [68]. Therefore, the collection of additional experimental data and resolved structures across a diverse range of antigens with respect to CATH classifications and sizes is in strong demand. Consequently, additional data can provide better training for BCE prediction methods and allow for the generation of larger independent test sets that will improve upon this review and establish even greater confidence. Thus, the successes and failures of computational BCE prediction can be better surveyed and improved accordingly.

## Figures and Tables

**Table 1:** Binary classifier evaluation metrics.

$Precision=\frac{TP}{TP+FP}$
$Recall=True\;Positive\;Rate(TPR)=\frac{TP}{TP+FN}$
$False\;Positive\;Rate(FPR)=\frac{FP}{FP+TN}$
$F1-Score=\frac{2*Precision*Recall}{Precision+Recall}$
$MCC=\frac{TP*TN-FP*FN}{\sqrt{\left(TP+FP)(TP+FN)(TN+FP)(TN+FN\right)}}$

**Table 2:** Comparison of <F1-score>, <MCC>, ROC-AUC, and PR-AUC determined on the test set with the surveyed computational methods.

	ISPIPab	VORFFIP	DiscoTope 2.0	DiscoTope 3.0	EPSVR	Spatom	SEPPA 3.0	BepiPred 3.0	Epitope3D
<F1-score>	0.312 ± 0.16	0.192 ± 0.18	0.133 ±0.17	0.241 ± 0.16	0.162 ± 0.21	0.161 ± 0.17	0.179 ± 0.14	0.241 ± 0.16	0.109 ± 0.10
<MCC>	0.230 ± 0.17	0.090 ± 0.18	0.017 ± 0.19	0.143 ± 0.17	0.054 ± 0.23	0.048 ± 0.19	0.067 ± 0.18	0.145 ± 0.17	−0.015 ± 0.09
ROC-AUC	0.77	0.66	0.47	0.75	0.56	0.64	0.65	0.71	0.59
PR-AUC	0.23	0.14	0.07	0.20	0.09	0.13	0.12	0.16	0.08

## Supplementary Materials

The following Supporting Information can be downloaded at: https://www.mdpi.com/article/10.3390/biom14080983/s1. The PDB IDs of the bound as well as the analogous unbound 29 test set antigens is given in the Supplementary Table S1, which also includes the total number of epitopic residues and the corresponding residue numbers of these epitopes in the unbound antigens.
