# Supplementary material for: Computational Methods to Predict Conformational B-Cell Epitopes
Source: Biomolecules. 2024 Aug 10;14(8):983. doi: 10.3390/biom14080983 (PMC11352882; doi:10.3390/biom14080983)
Supplement: Supplementary file 1 [file biomolecules-14-00983-s001.zip › biomolecules-3123025-supplementary.pdf]

Table S1: PDB IDs of bound antigens on the test set along with the PDB IDs of analogous unbound antigens, total number of epitopic residues on the unbound antigen and the residue numbers of these residues.

Bound_Antigen_PDB_ID	Unbound_Antigen_PDB_ID	Number of Epitope Residues	Epitope Residue Numbers on the Unbound Antigen
7DUO	2EF1	18	120+121+135+139+141+142+202+203+205+236+237+239+241+272+273+274+275+276
5BO1	2VJ2	18	190+201+202+203+204+205+206+207+209+211+219+221+222+224+246+247+250+252
3O2D	1WIO	18	3+94+96+120+122+123+124+125+126+127+140+141+142+162+163+164+165+166
6P50	3UP1	15	52+54+55+59+61+62+84+85+86+88+93+94+95+98+100
4RRP	1WG3	23	17+18+19+20+21+22+23+24+27+28+30+32+35+67+68+69+70+73+77+78+79+80+82
3LDB	3K2O	13	53+55+57+58+61+62+65+158+291+319+320+322+323
7LFA	7L6K	8	124+125+127+128+130+132+133+137
5DHV	6BSY	13	11+14+15+18+21+22+48+51+55+58+59+62+63
5H35	5WTR	21	1+2+5+6+9+55+57+59+62+107+110+111+113+114+115+164+168+169+172+173+176
4KUC	4Q2V	13	53+92+98+99+101+102+105+106+109+112+113+114+116
4CMH	6EDR	25	75+76+79+107+108+110+111+112+113+114+115+116+117+118+119+148+150+194+195+196+198+199+201+233+234
3PGF	5EDU	23	195+198+199+200+201+203+206+218+367+409+410+413+416+417+429+432+433+435+436+437+438+439+440
4JR9	4U4W	11	12+15+16+17+19+24+25+27+28+29+32
4OII	4OIE	21	232+235+236+237+239+240+253+256+257+258+260+261+262+264+265+289+293+294+314+315+351
3LEV	3LES	9	107+108+109+110+111+112+113+116+244
5CBA	7JNY	14	20+23+24+26+27+28+29+30+48+50+61+64+65+68
3MXW	3M1N	21	44+45+87+123+125+126+131+132+133+134+135+136+147+153+155+174+176+177+178+179+180
3NH7	2K3G	21	43+60+62+64+67+74+77+78+79+80+81+84+86+90+91+92+93+94+95+96+97
5JQ6	1N67	21	408+428+429+431+432+433+434+460+463+464+465+466+467+468+473+477+479+481+510+512+518
4ETQ	4ETQ	22	5+39+40+41+44+174+175+176+177+178+179+204+205+215+217+219+220+221+223+224+226+227
3KS0	1LDC	10	30+63+64+65+66+67+68+69+70+72
3LD8	6MEV	13	53+55+57+58+61+62+65+158+291+319+320+322+326
3WKM	3WKL	24	116+117+161+162+165+195+198+200+207+215+216+242+246+250+276+277+278+279+280+281+282+283+288+289
5FB8	1I16	19	38+39+40+42+43+44+45+46+51+53+63+65+66+94+95+96+97+99+102
2JEL	1HDN	15	1+2+3+4+34+41+64+66+67+68+70+71+72+75+76
2VXQ	1BMW	15	32+34+39+40+41+43+65+67+68+75+76+77+78+79+80
4U6V	6U49	16	173+179+181+182+183+185+186+187+188+189+190+191+200+263+264+266
3LIZ	1YG9	18	199+200+201+232+233+234+235+236+237+248+250+251+253+254+256+257+270+272
5MES	5KU9	12	174+205+208+308+309+310+311+313+317+318+320+322
